# Improving Prefrontal Oxygenation and Cardiac Autonomic Activity Following Meditation: A Functional Near-Infrared Spectroscopy (fNIRS) Study

**DOI:** 10.7759/cureus.65978

**Published:** 2024-08-01

**Authors:** Sushanta Mohanty, Deepeshwar Singh, Amit Singh, Dwivedi Krishna, Subarana Mohanty, Suhas Vinchurkar

**Affiliations:** 1 Division of Yoga and Life Sciences, Swami Vivekananda Yoga Anusandhana Samsthana, Bengaluru, IND; 2 Department of Yoga, Babasaheb Bhimrao Ambedkar University, Lucknow, IND; 3 Neuroimaging Instrumentation, Magstim EGI, Eugene, USA

**Keywords:** heart rate variability, prefrontal cortex, hemodynamic responses, brain imaging, meditation

## Abstract

Objective: The empirical evidence explicitly demonstrates that meditation practice enhances both brain functions and mental well-being. A meditative relaxation approach called the mind sound resonance technique (MSRT) has shown promising effects on children, adolescents, and people with psychological illnesses. This study aimed to investigate the effects of MSRT practice on brain hemodynamics, heart rate variability (HRV), mindfulness, and anxiety levels in college students.

Methods: Fifty volunteers in all genders (females, n = 30; males, n = 20) aged between 19 and 30 years were chosen from an educational institute and allocated into two groups, i.e., MSRT (n = 25) and supine rest (SR; n = 25). Enrolled participants were measured cerebral hemodynamics and HRV before, during, and after the MSRT or SR practice. The self-reported assessments including state anxiety and mindfulness were assessed before and after the intervention.

Results: The results demonstrated that practicing MSRT significantly improved oxygenation (p < 0.05) in the right prefrontal cortex (PFC) and increased low-frequency (LF) (p < 0.05) and decreased high-frequency (HF) (p < 0.05) component of HRV when compared to the baseline. The between-group analysis showed a significant difference between MSRT and SR in the standard deviation of the normal-to-normal (SDNN) (p < 0.05) component of HRV.

Conclusion: These crumbs of evidence imply that MSRT sessions may foster the development of anxiety-related coping skills by elevating mindfulness, promoting PFC oxygenation, and modulating HRV in MSRT practitioners.

## Introduction

The prefrontal cortex (PFC) is a crucial part of the brain located at the front of the frontal lobe. It is engaged in controlling impulsive or improper behavior. The PFC supports people in reining in their urges and making choices that take the long term into account [1]. In addition to controlling impulses, PFC plays a role in cognitive functions and emotional regulation. Furthermore, it facilitates executive processes, including organizing, resolving issues, and making choices [2]. The PFC, particularly the ventromedial and dorsolateral regions, plays a key role in emotional regulation [3,4]. Emotional regulation, in turn, affects autonomic nervous system activity, influencing heart rate and heart rate variability (HRV) [5]. Activities that engage the PFC, such as mindfulness meditation and cognitive reappraisal, have been associated with increased HRV [6,7]. Few investigations on meditation have demonstrated encouraging effects on the autonomic nervous system (ANS) as assessed by HRV. A study demonstrated that a brief chanting of Om increases the high frequency (HF) power of HRV, which is linked to parasympathetic activity, suggests relaxation, and promotes tranquility [8]. On the other hand, focused meditation increases low-frequency (LF) power and decreases HF power, and meditation without focusing had opposite results [9]. According to a recent study, during mindful breathing meditation, there was an increase in the autonomic balance index as measured by the HRV metrics of the root mean square of successive differences (RMSSD) and standard deviation of RR intervals (SDRR) [10]. These HRV studies imply that meditation practice may promote beneficial subjective changes in attention focus, happiness, and tranquility and the development of a coping mechanism for stressful situations [11,12]. It also appears to support "sympathovagal balance" [13].

Meditation practices, particularly mindfulness meditation, have been associated with changes in brain regions, and the PFC is one of the areas that has received considerable attention in research [14-16]. It has been found that regular meditation can lead to an increase in the thickness of the PFC, which is associated with improved cognitive abilities [17]. In addition, studies have shown that meditation can enhance the connectivity between the PFC and other brain regions, strengthening neural networks involved in emotional regulation and self-awareness [18]. These findings highlight the potential of meditation as a tool for promoting brain plasticity and overall mental well-being.

The mind sound resonance technique (MSRT) is a sound-based meditation method that creates resonance with mantras. Previous research [19] found that the MSRT improved cognitive performance, which includes sustained attention, focus, visual scanning, quick reaction activation and inhibition, psychomotor speed, mental flexibility, and information processing speed. In addition, it can be used to lower blood pressure, heart rate, tension, anxiety, and sadness while enhancing well-being, focus, willpower, relaxation, and self-esteem [20,21]. The mantras that are employed in the MSRT are used to produce resonance, which is then used to induce profound relaxation for both the mind and body. Despite mounting evidence of the MSRT's positive effects on mental and physical well-being, little is known about how it affects neurophysiology. Hence, the present study was planned to evaluate the effect of the MSRT on the PFC and HRV among young students.

The PFC is essential for decision-making, problem-solving, and attention, which affect mental health and academic success. Increasing prefrontal oxygenation may improve cognitive ability and college learning. In this work, prefrontal oxygenation and cardiac autonomic activity are linked, providing a comprehensive view of cortical and heart processes. The new merging of these variables can help better comprehend college students' well-being. To the best of our knowledge, this may be the first study to examine the impact of the MSRT on the PFC in order to evaluate oxygen levels and heart function in college students.

## Materials and methods

Participants

A total of 74 participants were screened, and out of that, 50 healthy participants of both genders (30 female), with a mean age of 23.0 (SD = 3.3 years), were recruited. All participants were recruited from Swami Vivekananda Yoga Anusandhana Samsthana (S-VYASA) University, an educational institute in Bengaluru, south of India. Table 1 shows the demographic details of the recruited participants in the present study.

**Table 1:** Demographic details of the participants. MoCA: Montreal Cognitive Assessment, GHQ12: 12-Item General Health Questionnaire, fNIRS: functional near-infrared spectroscopy, ECG: electrocardiogram, STAI: State Anxiety Inventory, MAAS: Mindful Attention Awareness Scale

Variables	Number of participants		Gender (in number)		Age (years) (M±SD)	Education (in number)	
	MSRT	SR	Male	Female		Graduation	Master
MoCA	25	25	20	30	23.76±3.30	8	42
GHQ12	25	25	20	30	23.76±3.30	8	42
fNIRS	21	17	15	23	24.05±3.59	6	32
ECG	20	16	15	21	24.03±3.44	6	30
STAI	23	23	18	28	23.87±3.30	7	39
MAAS	23	23	18	28	23.87±3.30	7	39

These participants were screened using the Montreal Cognitive Assessment (MOCA) and General Heath Questionnaire (GHQ-12). In addition, the participants who had (i) a minimum of six months experience in yoga, (ii) were able to read and write English, (iii) were physically and mentally fit to participate, and (iv) were willing to participate were included. The participants were excluded if (i) they had an allergy to applying spirit to the frontal head, (ii) had any kind of regular medication, and (iii) were smokers, drug addicts, and alcoholics. The eligible 50 participants were randomly allocated into two groups, i.e., MSRT (n = 25; 14 female) and supine rest (SR; n = 25; 16 female) using Research Randomizer (https://www.randomizer.org/). The signed informed consent form was obtained from each participant after explaining the assessment procedures in the study.

Design of the study

**Figure 1:**
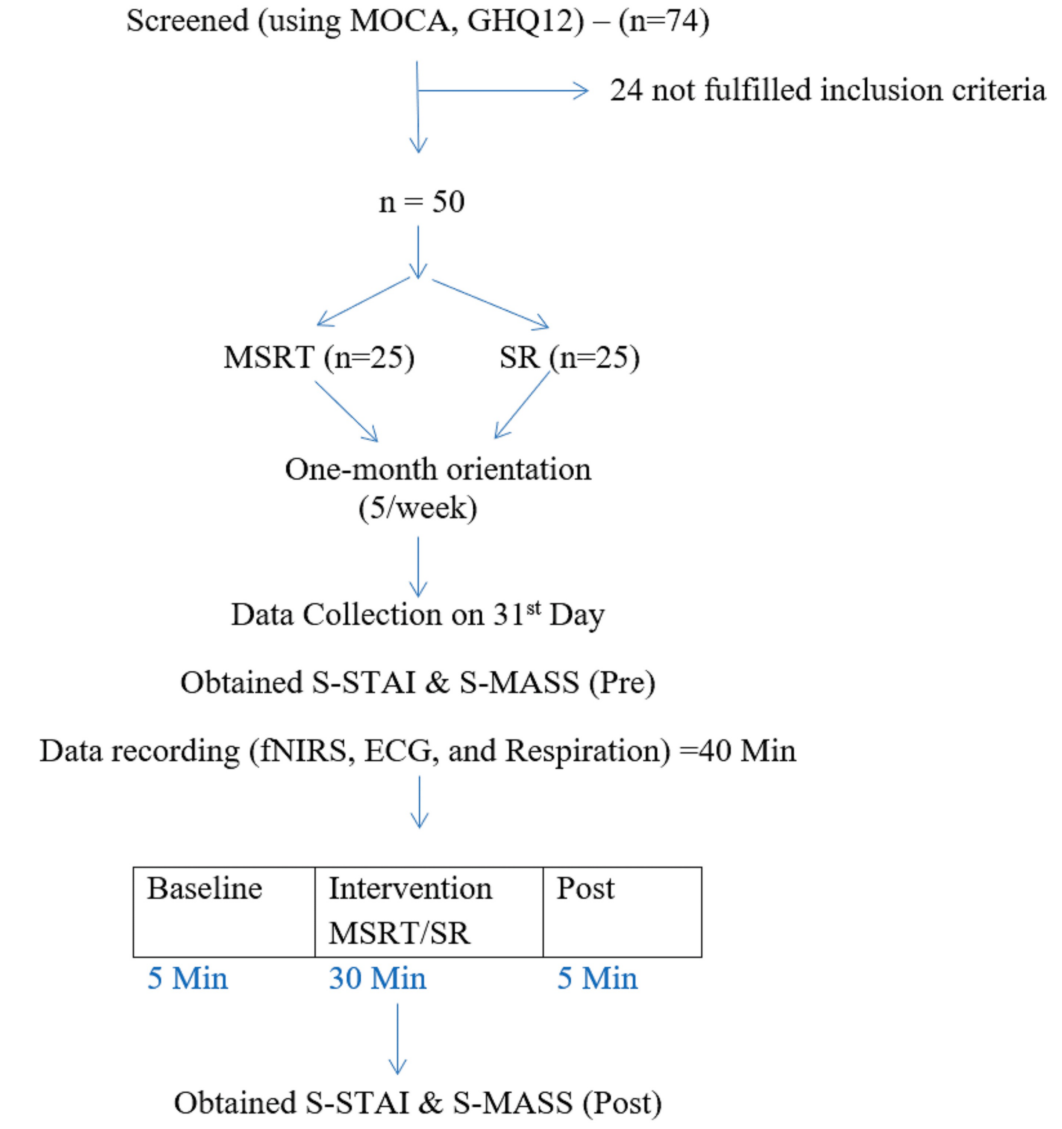
Schematic presentation of the participants' recruitment and experimental protocol.

The research included participants from September 2021 to March 2022. Over one one-month orientation program, the experimental group practiced the MSRT while the control group rested supine. An experienced meditation instructor led these 30-minute orientation programs for five days a week. The purpose of the orientation program was to maintain uniformity in the practice. The practice of the MSRT included the chanting of Maha Mrityunjaya Mantra, A Kara, U Kara, M Kara, A-U-M Kara, Ajapa Japa OM, silence, resolve, and closing prayer. The control group rested in the supine position for the same duration. After the one-month orientation program, the participants underwent assessments for frontal hemodynamic activity, HRV, respiration, mindfulness, and anxiety. The participants were tested for frontal hemodynamic activity, HRV, mindfulness, and anxiety after the orientation program. MSRT or SR sessions were recorded at baseline (BS), during practice (MSRT/SR), and post-practice. The schematic presentation of the study design is shown in Figure 1.

Assessments

The participants were assessed for brain hemodynamics, cardiac activity, and self-reported scales as described below.

Functional Near-Infrared Spectroscopy (fNIRS)

**Figure 2:**
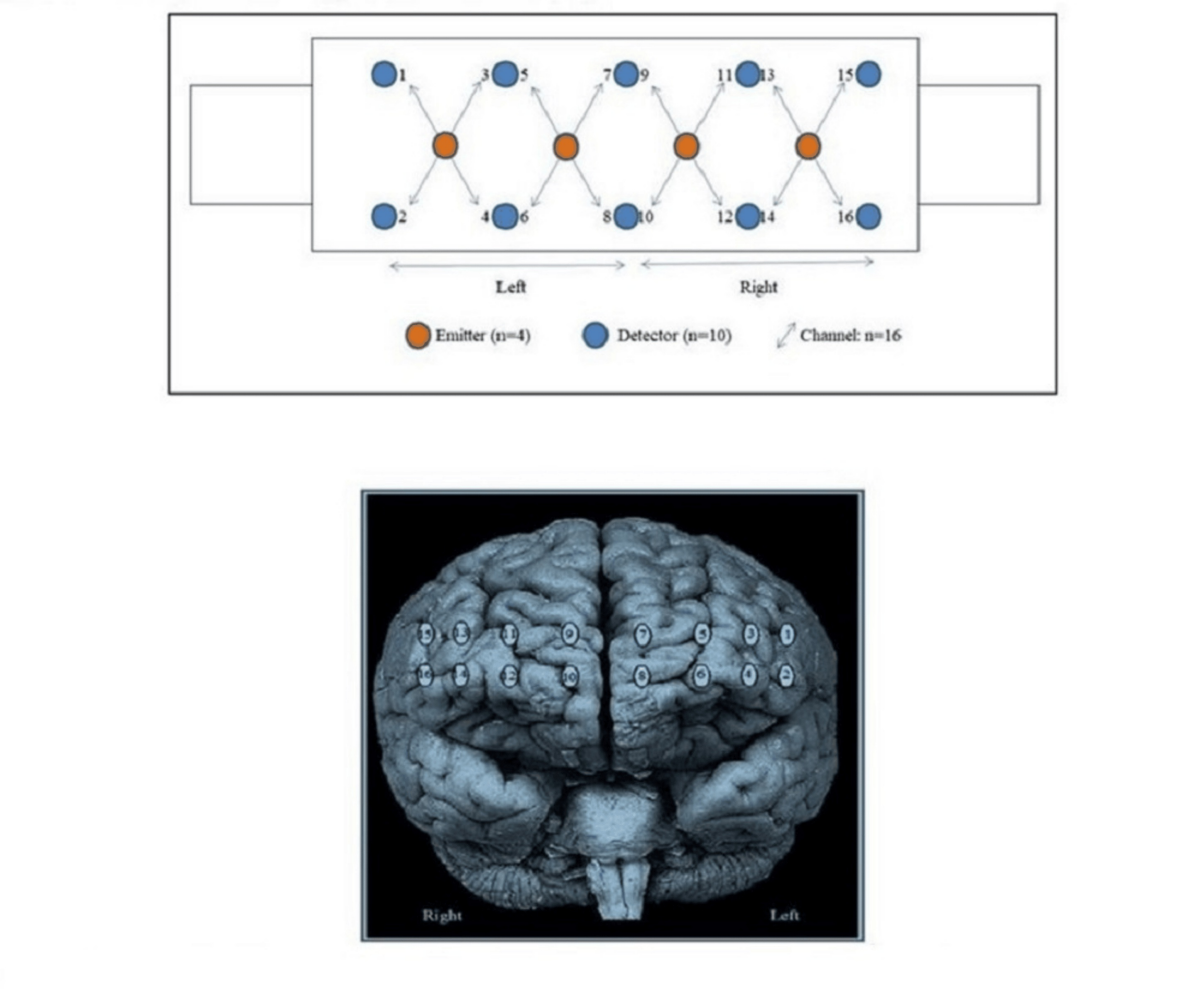
Infrared emitter and probe configuration (2a) for functional near-infrared spectroscopy. fNIRS opted measurement location on the prefrontal cortex (2b). fNIRS: functional near-infrared spectroscopy

The fNIRS is a non-invasive imaging technique that measures brain activity by detecting changes in blood oxygenation and blood flow in response to neural activity. The oxygen content in the frontal brain region was monitored with the use of a 16-channel continuous-wave near-infrared imager system (FNIR1000-ACK-W, BI-OPAC Systems, Inc., USA). A sampling rate of 2 Hz was used by default [22]. An analytical software and stimulus presentation platform that uses near-infrared spectroscopy (nirsLAB; V201904) measures to analyze, visualize, and display data; fNIR Imager & COBI Studio software (Drexel University, Philadelphia, PA, 2018) was used to acquire the optical imaging data. As shown in Figure 2, four LED diodes of 730 nm, 830 nm, and 850 nm were used to provide light and 10 photodiodes served as detectors, which were arranged symmetrically in an area of 3.5 x 14 cm^2^. This resulted in 16 nearest source-detector channels separated at 2.5 cm as indicated in the previous study [14].

A sensor pad was placed on the subject's forehead. The hair of the subject was taken care of, and if necessary, hair bands were used to hold the hair in place. Before taking data, we checked that the wires on the sensor pad had been fixed properly. The cord locks were used with the cloth straps, and we adjusted the tightness so that the subject was comfortable while wearing the harness. In addition, we checked that the headband is tight and the sensor makes good contact with the forehead, but it should not be too tight in order not to restrict blood flow. According to previous research, a black cap was placed over the subject's head to block out extraneous light [23].

HRV

HRV refers to the variation between consecutive heartbeats. HRV is regulated by the sympathetic and parasympathetic nervous systems and other controls, and thus, HRV serves as a quantitative indicator of the heart's autonomic control (European Society of Cardiology and American Society of Pacing and Electrophysiology, 1996). The ECG was assessed throughout a session that lasted 40 minutes (baseline: five minutes, during the intervention: 30 minutes, and post-resting: five minutes) using a 16-channel polygraph system (MP 100 BIOPAC, AcqKnowledge software, BIOPAC System Inc., USA). As part of the ECG recording process, Ag/AgCl pregelled electrodes were used (Tyco Healthcare, Germany) and the standard limb lead II configuration was utilized with a 1024 sampling rate [24].

Respiration Rate

To address the potential influence of respiration on the HRV, particularly concerning lower frequencies (approximately 0.1 Hz), respiration was monitored with HRV simultaneously. The respiratory rate was captured using a volumetric pressure transducer positioned around the trunk, situated approximately 8 cm below the lower costal margin, while the participants maintained an upright seated posture. This approach aimed to account for respiratory effects and isolate HRV variations more accurately during data analysis.

State Anxiety Inventory (STAI-Y1)

A state anxiety assessment was conducted using Spielberger's State Anxiety Inventory (STAI; Form Y-1) [25]. The STAI is considered to be an accurate, reliable, sensitive, and valid instrument with a high degree of internal consistency, with a Cronbach's alpha of 0.83 for the total score. An average score was calculated using a four-point Likert scale, with each item being scored from 1 to 4. A total of 10 items out of 20 were considered reverse scores. An interval of 20 to 80 was used for scoring [26]. Indicative of heightened levels of present anxiety or emotional distress are State Anxiety Scale scores that are higher on the STAI. Greater physiological arousal, heightened tension, apprehension, and concern may be experienced by those who obtain higher scores on the State Anxiety Scale when confronted with stressful or difficult circumstances.

Mindful Attention Awareness Scale (MAAS)

The MAAS is a five-item measure of mindfulness's short-term or current manifestations, namely, a receptive state of mind. Participants are aware of what is happening in the present [27]. The participants have requested to provide responses that reflect what they have experienced rather than what they think generally. The questionnaire included the following: "I was having difficulty maintaining focus on what was happening"; "I was doing something without paying attention"; etc. They were asked to choose any one of the options between "Not at all" to "Very much so." A seven-point Likert scale was used for the calculation of the average score, and each item was scored on a scale ranging from 0 to 6. A reverse score was applied to each item before an average value was calculated for each item. The higher the scores are, the more mindfulness is reflected in the state of the person.

Orientation program

The orientation program of the MSRT and SR was given to the recruited participants for one month (30 minutes/day).

The MSRT is an advanced yoga technique that was devised by Dr. H R Nagendra, Chancellor of S-VYASA (Deemed to be University), Bengaluru, with the aim of attaining profound relaxation [28]. The MSRT entails perceiving the internal vibrations and resonance generated during the recitation of the Mahamritunjaya mantra and the syllables A, U, M, Om, and Om with closed eyes. A person practicing MSRT experiences body-wide resonance when chanting aloud (Ahata: heard) or in their mind (Anahata: unheard). This is achieved by alternating the forms Ahata "A" and Anahata "A" three times. This is then repeated in an identical fashion for every other chant. The internal vitality of the body is revitalized by the resonance produced by the MSRT. It penetrates deeper strata of silence and protects against all anxieties and fears. It can result in a profound sense of relaxation and expansion, which is the foundation of the therapeutic properties of these traditional chants known as mantras.

Deep levels of body and mind relaxation associated with these mindfulness practices can lower sympathetic nervous system activation, raise parasympathetic nervous system activity, and reestablish homeostasis [20,29].

In the SR group practice, the participants were asked to lie down on their backs with closed eyes for 30 minutes [30].

Procedure of data collection

All the participants were asked to complete the STAI-Y1 and MAAS questionnaires to assess their anxiety state and mindfulness before and after the practices (MSRT and SR). The participants were instructed to sit upright comfortably on a chair in a light-dampening and sound-attenuating chamber. The continuous hemodynamic changes of the PFC were monitored through fNIRS and the ECG during baseline (five minutes), during practice (30 minutes), and post (five minutes).

Data preprocessing

The fNIRS signal preprocessing was carried out using NirsLab V.201904 (NIRx Medical Technologies, 15 Cherry Lane, Glen Head, NY 11545). A digital bandpass filter was applied to the original optical density signals in the range of 0.01-0.2 Hz. A modified Beer-Lambert equation [31] was used to calculate the relative change curves for oxyhemoglobin (HbO_2_) and deoxyhemoglobin (HbR). Lastly, data on HbO_2_ and HbR relative concentration signals were obtained [31]. Averaging signals from channels 1 through 8 yielded left prefrontal cortex (LPFC) activity known as the left hemisphere (LH) of the PFC. Channels 9 through 16 yielded right prefrontal cortex (RPFC) activity known as the right hemisphere (RH) of the PFC. Baseline relative change was compared with during practice and post-state. Due to the bad channel and artifacts in the NIRS signals, data of four participants from the MSRT group (female, n = 01; male, n = 03) and eight participants from the SR group (female, n = 06; male, n = 02) were eliminated during extraction, and noise-free data (MSRT, n = 21; SR, n = 17) were carried out for further analysis.

Similarly, ECG and respiration data were extracted using AcqKnowledge 4.1 and Kubios HRV software. Data were visually inspected offline, and noise-free data were included for further analysis. The absence of abnormal beats, movement artifacts, or baseline drift impacting the ECG trace was confirmed. Due to the poor signal quality in the ECG and respiration waveform, the data of five participants from the MSRT group (female, n = 02; male, n = 03) and nine participants from the SR group (female, n = 07; male, n = 02) were rejected during extraction, and noise-free data (MSRT, n = 20; SR, n = 16) were carried out for further analysis.

Sinus arrhythmia is a natural variation in heart rate that occurs during the breathing cycle. Typically, the heart rate increases during inhalation and decreases during exhalation. This phenomenon is known as respiratory sinus arrhythmia (RSA) and is considered a normal physiological response, especially prominent in younger individuals and those with higher cardiovascular fitness. However, in the current study, we utilized a respiratory rate of 6 to 8 breaths per minute, which may mitigate the respiratory effect on HRV outcomes.

Fast Fourier transformation analysis was applied to obtain the HRV power spectrum. The examination of the HRV series encompassed investigations into the frequency domain: (i) low-frequency (LF) band (0.05-0.15 Hz), (ii) high-frequency (HF) band (0.15-1.50 Hz), and (iii) LF/HF ratio. The low-frequency and high-frequency band values were expressed as normalized units. The following components of the time domain of the HRV were analyzed: (i) mean RR interval (the mean of the intervals between adjacent QRS complexes or the instantaneous heart rate), (ii) RMSSD (square root of the squared difference of successive NN intervals, where NN = normal to normal intervals), and (iii) pNN50 (proportion derived by dividing NN50 by the total number of NN intervals, where NN50 is the number of interval differences of successive NN intervals greater than 50 ms) [32].

A total of four psychometric data (MSRT, n = 2; SR, n = 2) were excluded due to incomplete or inaccurate responses, and 46 data (MSRT, n = 23; SR, n = 23) were included for further analysis.

Statistical analysis

The data were exported to Microsoft Excel (Microsoft Corporation, USA), and statistical analysis was done in IBM SPSS Statistics for Windows, version 21.0 (released 2012, IBM Corp., Armonk, NY). An independent sample t-test tested the differences in the participant’s characteristics. The fNIRS and HRV parameters were assessed by repeated-measures analysis of variance (RM-ANOVA). The fNIRS parameters were assessed in the group (MSRT vs. SR) and served as the between-group analysis. In addition, the state of measurement (baseline, during practice, post) and hemisphere (left vs. right) were treated as within-participant variables. Similarly, the HRV parameters, assessed in the MSRT and SR groups, served as the between-group factor, with the state of measurement (baseline, during practice, and post) also considered as a within-participant variable. Post-hoc Bonferroni adjustments were assessed for the fNIRS and ECG data. Normality test was used for psychological data STAI (Y1) and MASS. The STAI score was normally distributed, and a paired sample t‑test was performed. The MAAS score was not normally distributed; hence, the Wilcoxon signed-rank test was performed. The level of statistical significance for all comparisons was set as p < 0.05.

## Results

fNIRS

RM-ANOVA with the two groups (MSRT vs. SR) x two hemispheres (left hemisphere vs. right hemisphere) x three states (baseline vs. intervention, intervention vs. post, and baseline vs. post) were used to examine the HbO_2_ and HbR data. The findings indicated that there was no significant interaction effect in HbO_2_, while the interaction for state x hemisphere was significant in HbR (F(2,72) = 3.12, p = 0.05, ηp2 = 0.08).

The HbO_2_ and HbR analysis findings of the post-hoc analysis with Bonferroni correction showed that there was a statistically significant increase in HbO_2_ in the RH relative to the LH during the intervention (p < 0.001) and post-intervention (p < 0.05), respectively.

Similarly, a significant decrease of HbR in the RH compared to the LH at the baseline of the MSRT group (p < 0.05). Moreover, a significant change in HbO_2_ (p < 0.05) and HbR (p < 0.05) in states was observed, between the baseline and during the MSRT practice in the RH (Table 2). However, there was no significant change in the cerebral oxy-Hb variation in the control group. There was a trend of continuous improvement in the HbO_2_ during the sessions observed in the right PFC, as shown in Table 2.

**Table 2:** Repeated measures of ANOVA with Bonferroni test describes the blood volume (µMol) Mean ± SD, left vs. right hemisphere, states (S1, S2, and S3). N= MSRT (21), SR (17). ^#^ p < 0.05; ^## ^p < 0.001. * indicates within-state comparison (S1 to S2) and # indicates between-hemispheric comparison (left hemisphere vs. right hemisphere).

		Left hemisphere			Right hemisphere		
		Baseline (S1)	Intervention(S2)	Post (S3)	Baseline (S1)	Intervention(S2)	Post (S3)
Description	Group	MEAN±SD	MEAN±SD	MEAN±SD	MEAN±SD	MEAN±SD	MEAN±SD
HbO2 (uMol)	MSRT	33.7±128	24.5±32.0	2.98±69.0	11.1±148	84.9±57.9*##	144±347#
	SR	23.8±54.5	38.0±38.6	23.8±52.4	14.2±106	67.5±60.4	37.0±72.7
HbR (uMol)	MSRT	11.6±69.1	2.77±14.9	-16.3±60.5	-44.6±115#	22.6±41.3*	-0.415±117
	SR	4.83±42.9	15.4±29.9	12.0±38.0	-5.49±73.7	-1.86±45.0	26.0±48.8

HRV

The RM-ANOVA results for the measures of frequency domain analysis showed a significant main effect in LFnu (F(1.63, 55.45) = 4.51, p<0.05, ηp2 = 0.12), HFnu (F(1.64, 55.58) = 4.55, p < 0.05, ηp2 = 0.12), and LF/HF (F(1.81, 61.62) = 4.02, p < 0.05, ηp2 = 0.11). In the measures of the time-domain analysis, there was no significant main effect in the variables.

Post-hoc analysis with Bonferroni correction in the frequency-domain analysis showed a significant increase in LFnu (between S1 and S2) (p < 0.05) and HFnu (between S2 and S3) (p < 0.01) and a decrease in the LF/HF ratio (between S2 and S3) (p = 0.001), LFnu (between S2 and S3) (p < 0.01), and HFnu (between S1 and S2) (p < 0.05) during the MSRT sessions. Similarly, time-domain measures showed a significant increase in the SDNN during the intervention (S2) in the MSRT group compared with SR. However, there was no discernible change in the frequency domain and time domain for the SR group (Table 3). Moreover, respiration data did not show any significant changes and differences in either group.

**Table 3:** HRV measures at baseline, intervention, and during post-session. Values are grouped as mean±SD, mean difference, and percentage change (%). Pairwise comparison between states: S1 vs. S2, S1 vs. S3, S2 vs. S3. * indicates the significance level at p < 0.05 (between S1 and S2); *$$* and *$$$ *indicate the significance level at 0.01 and 0.001, respectively (between S2 and S3); and # indicates the significance level at 0.05 between the MSRT and SR sessions. Variable and initials used: Mean RR: mean RR interval (ms); SDNN: standard deviation of normal to normal RR intervals (ms); RMSSD: root mean square standard deviation, pNN50: LF/HF ratio, nu-normalized units; LF: low frequency, HF: high frequency, nu: normalized units. S1: baseline, S2: during intervention, S3: post.

		Mean±SD				Mean difference			% change			
	Variable		Baseline (S1)	Intervention (S2)	Post (S3)	S1-S2 (BS-Int)	S1-S3 (BS-Post)	S2-S3 (Int-Post)	S1-S2 (BS-Int)	S1-S3 (BS-Post)	S2-S3 (Int-Post)	
Time domain	Mean RR	MSRT	764±88.3	756±109.66	755±92.12	-7.96	-8.41	-0.45	-1.04	-1.10	-0.06	
		SR	757±76.97	761±71.45	729±91.09	3.80	-27.98	-31.78	0.50	-3.69	-4.18	
	SDNN	MSRT	127±130.35	127±75.51#	107±71.42	-0.14	-19.72	-19.58	-0.11	-15.55	-15.46	
		SR	86±59.77	83±40.00	98±63.08	-3.37	12.01	15.38	-3.91	13.90	18.53	
	RMSSD	MSRT	154±188.51	141±113.87	127±105.28	-12.30	-26.78	-14.48	-8.01	-17.44	-10.25	
		SR	90±86.35	85±60.84	100±89.37	-4.25	11.08	15.34	-4.75	12.36	17.96	
	pNN50	MSRT	36±28.69	33±25.50	32±26.03	-2.76	-4.26	-1.49	-7.72	-11.90	-4.53	
		SR	28±22.86	26±19.01	28±24.84	-2.06	0.31	2.37	-7.30	1.08	9.05	
Frequency domain	LF/HF	MSRT	1.40±1.76	2.67±2.78	1.15±1.07^$$$^	1.27	-0.25	-1.52	90.63	-17.89	-56.93	
		SR	1.61±1.49	1.46±0.98	1.75±1.28	-0.15	0.14	0.29	-9.50	8.61	20.01	
	LFnu	MSRT	48.42±16.38	57.45±17.62*	45.99±17.19^$$^	9.03	-2.43	-11.46	18.65	-5.02	-19.95	
		SR	53.31±17.88	51.98±14.34	56.63±17.19	-1.33	3.32	4.65	-2.50	6.23	8.95	
	HFnu	MSRT	51.29±16.33	42.31±17.45*	53.68±17.06^$$^	-8.98	2.39	11.37	-17.51	4.66	26.87	
		SR	46.39±17.69	47.78±14.31	43.12±17.03	1.38	-3.28	-4.66	2.98	-7.06	-9.75	

Self-reported questionnaires

**Figure 3:**
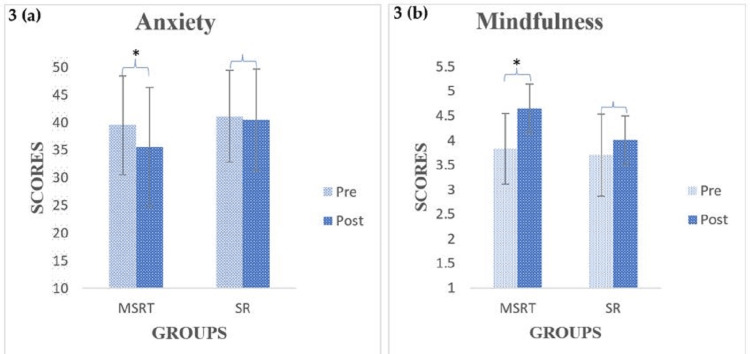
Within-group outcomes of anxiety and mindfulness for the MSRT and SR groups. Paired-sample t-test scores for anxiety (3a) and Wilcoxon signed-rank test scores for mindfulness (3b) are shown in the bar graph, where * indicates a significant level at p < 0.05. MSRT: mind-sound resonance technique, SR: supine rest

The results of the STAI showed a significant decrease in the anxiety score from pre to post (p < 0.05) in the MSRT, while no significant change was found in the SR group, as shown in Figure 3a. Similarly, the results of the MAAS showed a significant improvement in mindfulness from pre to post (p < 0.05) in the MSRT, while no change was found in the SR group, as shown in Figure 3b. However, there was no significant difference between the MSRT and SR groups (p > 0.05) at post-intervention. An independent sample test was performed on demographic data and did not find significant differences between the groups at p < 0.05.

Correlation

**Figure 4:**
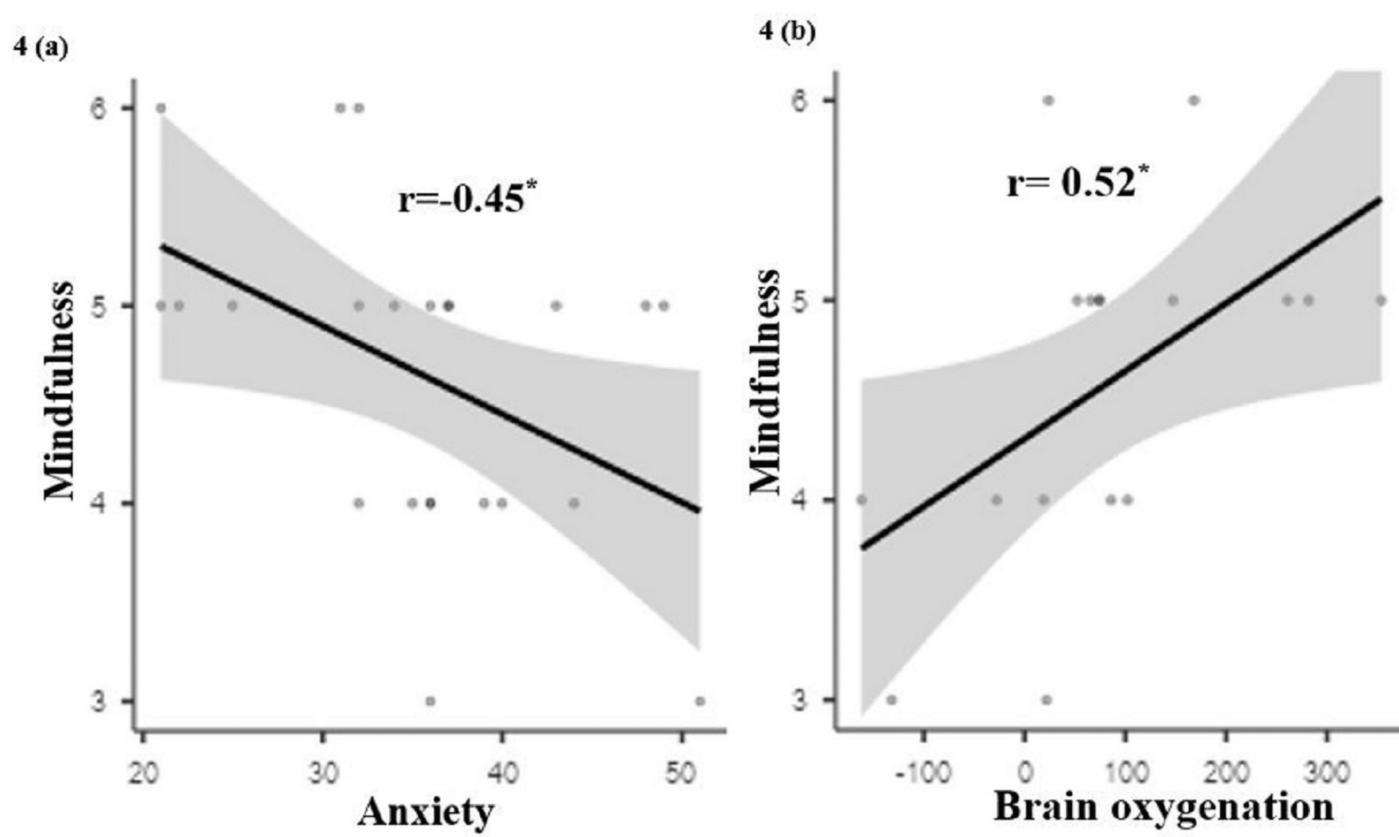
Graphical representation of mindfulness with anxiety and brain oxygenation. 4a indicates the correlation between mindfulness and anxiety, and 4b indicates the correlation between mindfulness and brain oxygenation (HbO_2_).

Figure 4(a) illustrates Pearson's correlation, which indicates that mindfulness decreases with increasing anxiety (r= -0.45; p < 0.05). According to Figure 4b, there is a positive relationship (r = 0.52; p < 0.05) between greater levels of mindfulness and enhanced cerebral oxygenation in the right hemisphere of the brain. There was no association between any of the other factors.

## Discussion

In this study, we aimed to investigate the effect of the MSRT and SR on prefrontal hemodynamic responses and cardiac autonomic activities in college students. The practice of MSRT showed improvement in cerebral oxygenation at the right PFC, which is similar to the results of mindfulness meditation [18]. The cardiac autonomic activity also showed a withdrawal of the vagal tone during the MSRT and increased vagal tone after the MSRT practice. These results suggest that the focus needed for the MSRT may increase sympathetic activity. This is the body's response to the increased awareness and attention that come from focused meditation [33]. Meditation has the potential to bring about a condition of alert rest because it helps the mind become more focused and quiet. Following meditation, if practitioners choose to concentrate on any activity or work, it is possible that this will not have an effect on the sympathetic activity of the autonomic nervous system [34]. However, few meditation practices demonstrated an increase in HF power, which indicates that the vagal tone and other research revealed the opposite outcomes. The primary cause of these inconsistent findings is RSA. When the breathing rate falls below 0.15 Hz during meditation, the RSA component shifts from the high-frequency zone to the low-frequency region, which can lead to the overestimation and underestimation of both low- and high-frequency powers. Hence, fluctuations in the respiration rate could be an important component that may influence HRV analysis [35,36]. Consequently, even though the LF component increased initially during the MSRT practice, it might be linked to the vagal tone, which supports the autonomic nervous system and promotes a sense of calm and well-being [37].

Akselrod et al.'s 1981 work established the relationship between HRV frequency and autonomic nervous system activity. The HRV LF band (0.04-0.15 Hz) was linked with sympathetic modulation [38]. HRV analysis was further clarified by later research and the 1996 Task Force of the European Society of Cardiology and the North American Society of Pacing and Electrophysiology recommendations [39]. After the sympathetic activity was linked to LF power, parasympathetic activity was found to affect it, especially during vagal withdrawal. The present study showed significant changes in HF power (0.15-0.4 Hz), which predominantly indicates parasympathetic modulation, and the LF power showed significant changes during post, which regulates sympathetic activity marker (Task Force of the European Society of Cardiology, 1996). Considering that breathing rate, physical activity, and physiological parameters can affect LF power interpretation. Moreover, the physiological changes that are connected to variations in HRV parameters are crucial for improving alertness and cognitive performance [40].

Increased cerebral oxygenation ensures that the brain receives an adequate supply of oxygen, which is crucial for optimal brain performance [14]. In addition, heightened sympathetic activity can promote a state of increased arousal and focus, enabling individuals to better concentrate and respond to cognitive tasks. Thus, engaging in regular MSRT practice can have a positive impact on both mental and physical well-being [21].

The self-reported questionnaires showed a reduction in state anxiety and enhanced mindfulness among participants who followed the MSRT session compared to the SR, which is consistent with the earlier meditation study [41]. MSRT practice may have a component of mindfulness, which is a state of being present and aware of one’s thoughts, emotions, and sensations in the present moment. By incorporating mindful MSRT practice into their daily lives, individuals can develop a greater sense of self-awareness and reduce the negative effects of stress and anxiety. The reduction in state anxiety observed among participants suggests that the MSRT practice may serve as an effective tool for managing and alleviating symptoms of anxiety [42]. Moreover, the enhanced mindfulness reported by participants indicates that the MSRT practice can foster a greater sense of clarity and focus, allowing individuals to navigate through their daily lives with more ease and intention [43].

Overall, these findings highlight the potential benefits of incorporating the MSRT into one's routine for promoting mental and emotional well-being. Several studies support the outcome of the study that meditation can enhance cerebral oxygenation in the PFC [14,44]. One of the primary mechanisms through which cerebral oxygenation increases is focused attention on the breath and chanting mantras during the practice of MSRT. By regulating breath and practicing deep focused meditation, practitioners can increase the oxygen supply to the brain [45]. This conscious regulation of mindful activity not only improves oxygen delivery but also facilitates relaxation, activates the parasympathetic nervous system, and promotes a sense of calm [8]. Higher oxygenation in the right PFC was seen in the hemodynamic results during MSRT practice sessions, indicating that the MSRT practice may improve attentional and self-awareness functions. Previous studies on mindfulness meditation, which involves non-judgmental awareness of thoughts and sensations, have been associated with increased blood flow to the PFC [18]. This enhanced blood flow contributes to improved oxygenation, supporting optimal brain function. Imaging techniques like functional magnetic resonance imaging (fMRI) and near-infrared spectroscopy (NIRS) have helped us understand how the brain changes during meditation [46,47]. These studies consistently show increased activity and oxygenation in the PFC during and after meditation sessions. 

The relationship between meditation and cerebral oxygenation is not only evident in acute effects but also in long-term structural changes. Regular meditation practice has been linked to alterations in brain structure, including increased gray matter density in the PFC [18,48]. These structural changes may signify enhanced neural connections and more efficient use of oxygen and nutrients in this critical brain region.

Beyond the physiological aspects, the improvement in cerebral oxygenation through meditation aligns with cognitive benefits [49]. Studies have demonstrated that enhanced oxygen supply to the PFC is associated with improvements in attention, cognitive flexibility, and emotional regulation [42]. These cognitive enhancements can have far-reaching implications for HRV modulation, stress reduction, anxiety management, and overall cognitive performance following MSRT [50,51]. While the existing body of research provides compelling evidence for the positive impact of meditation on cerebral oxygenation in the PFC, further exploration is necessary to elucidate the specific mechanisms at play. In addition, considering the diversity of meditation practices, more research is needed to determine whether different techniques or durations of practice yield distinct effects on cerebral oxygenation. To the best of our knowledge, this is the first study in which college students performing MSRT had their autonomic activity and hemodynamic responses recorded concurrently.

Limitations

Although the results are positive, the current study includes a number of limitations. The use of an immediate effect design is the principal limitation of the study. This design has the potential to result in temporary effects of MSRT practice, which in turn restricts to generalize of the results. When it comes to the influence that the orientation practice of MSRT has on outcome variables, it is hard to disregard it. The study used plain SR as a control, and a better outcome would be expected with an active control. The placement of autonomic sensors and fNIRS sensor belts on the forehead during data collection may potentially have an impact on MSRT practice. It is possible that systemic physiological disturbances and low signal quality will have an effect on the NIRS recording because we did not employ the short separation channel. The scope of the study can be expanded to investigate the effects of MSRT on the hemodynamics of the brain and the regulation of the heart in college students over a longer period. In addition, cognitive tasks may be utilized to assess the extent to which enhanced oxygenation affects the PFC.

## Conclusions

The evidence thus far suggests a promising relationship between meditation and improved cerebral oxygenation in the PFC. This phenomenon not only sheds light on the physiological mechanisms underlying the cognitive benefits of meditation but also underscores the potential for meditation to be utilized as a tool for optimizing brain function and promoting mental well-being. As research in this field progresses, a deeper understanding of the intricate interplay between meditation, cerebral oxygenation, and cognitive function will likely emerge, offering valuable insights into both neuroscience and holistic approaches to mental health.
